# Smartphone and medical related App use among medical students and junior doctors in the United Kingdom (UK): a regional survey

**DOI:** 10.1186/1472-6947-12-121

**Published:** 2012-10-30

**Authors:** Karl Frederick Braekkan Payne, Heather Wharrad, Kim Watts

**Affiliations:** 1 King’s College Hospital NHS Foundation Trust, Denmark Hill, SE5 9RS, London; 2 School of Nursing, Midwifery & Physiotherapy, University of Nottingham, Nottingham, NG7 2HA, UK

**Keywords:** Smartphones, App use, Medical students, Doctors, Mobile technologies, Survey

## Abstract

**Background:**

Smartphone usage has spread to many settings including that of healthcare with numerous potential and realised benefits. The ability to download custom-built software applications (apps) has created a new wealth of clinical resources available to healthcare staff, providing evidence-based decisional tools to reduce medical errors.

Previous literature has examined how smartphones can be utilised by both medical student and doctor populations, to enhance educational and workplace activities, with the potential to improve overall patient care. However, this literature has not examined smartphone acceptance and patterns of medical app usage within the student and junior doctor populations.

**Methods:**

An online survey of medical student and foundation level junior doctor cohorts was undertaken within one United Kingdom healthcare region. Participants were asked whether they owned a Smartphone and if they used apps on their Smartphones to support their education and practice activities. Frequency of use and type of app used was also investigated. Open response questions explored participants’ views on apps that were desired or recommended and the characteristics of apps that were useful.

**Results:**

257 medical students and 131 junior doctors responded, equating to a response rate of 15.0% and 21.8% respectively. 79.0% (n=203/257) of medical students and 74.8% (n=98/131) of junior doctors owned a smartphone, with 56.6% (n=115/203) of students and 68.4% (n=67/98) of doctors owning an iPhone.

The majority of students and doctors owned 1–5 medical related applications, with very few owning more than 10, and iPhone owners significantly more likely to own apps (Chi sq, p<0.001). Both populations showed similar trends of app usage of several times a day. Over 24hours apps were used for between 1–30 minutes for students and 1–20 minutes for doctors, students used disease diagnosis/management and drug reference apps, with doctors favouring clinical score/calculator apps.

**Conclusions:**

This study found a high level of smartphone ownership and usage among medical students and junior doctors. Both groups endorse the development of more apps to support their education and clinical practice.

## Background

Smartphones have become ubiquitous among the general public. From internet to email, they offer *onthego* access to information never before possible. Within the healthcare population, the utilisation of smartphone and other mobile devices, such as the personal digital assistant (PDA) and handheld tablets, has the potential to have a positive impact upon patient care. Specifically, by providing personnel with immediate access to medical and health information, this technology can lead to improved decision-making and reduced numbers of medical errors [1,2], improved communication between hospital medical staff [3,4] and enhanced telemedicine capability [5,6].

The release of Apple’s *iPhone* in June 2007 (Apple Inc, Cupertino, CA, USA) was arguably the birth of the smartphone. It blended the attributes of the PDA with those of the mobile phone. From this point the market developed rapidly, with Apple recently releasing their *iPhone 4S* version, and competitors releasing equivalent models. From a healthcare perspective the pivotal moment was the launch of the Apple Appstore in July 2008. This gave phone users the ability to download a specific software application or “app” from an online shop. Rival companies such as Google Android and Blackberry have since followed suit with similar schemes. This represents an unrivalled ability to disseminate up-to-date information, at exceptional speed across a chosen population. Indeed to date there are a staggering 500,000 iPhone apps available to download [7].

In this regard the utilisation of smartphones by medical personnel has recently evolved. Doctors are now able to hold textbooks on their smartphone and use resources such as medical calculators and drug formularies. Moreover these apps update themselves, and are relatively easy to produce and release. While early literature focused on properties such as remote information access and improved communication, the recent trend has been for personnel or departments to use this technology to develop customised apps to improve an area of work. Visualisation of radiological images on smartphones, so-called ‘teleradiology’, has been a popular area of research [8-11], as have clinical guideline/decision support apps [12-14].

The smartphone has also proved useful within medical student populations. Trelease described the use of the smartphone as a potential “learn anywhere” resource for students [15], with further research exploring the use of podcasts on smartphones as a way of delivering education [16]. Within medical school the requirement of gaining competency sign offs during clinical attachments is very applicable to handheld technology [17], with evidence for improved case logbook use [18].

As a result of these developments the need for a study examining the uptake and application of smartphones was identified. The aims of the study were to: identify the extent to which junior doctors and medical students own smartphones and use them to enhance their clinical activities; and how often they use apps for education and clinical professional development. This survey was undertaken as a pilot study, to provide baseline data for future research being undertaken by the authors, planning to investigate a smartphone based hospital-linked app as an intervention to aid the clinical activities of junior doctors and medical students soon to qualify as junior doctors.

## Methods

We distributed 2 on-line questionnaires, via email, using a recognised survey website ( http://www.surveymonkey.com). Questionnaire 1 (Appendix A) was sent to Foundation level junior doctors (newly qualified doctors analogous to American ‘Intern’ position), years 1 and 2, working across one health region encompassing 8 hospitals and employing 601 Foundation level doctors. Questionnaire 2 (Appendix B) was a modified version of questionnaire 1; adapted to the different survey group to account for differences in participant role and environment. This survey was sent to medical students, years 1 to 5 in an East Midlands University in the United Kingdom (UK) within the same healthcare region, with 1,706 registered undergraduate medical students. The medical degree offered by this university comprises of 5 years total study, with students moving from pre-clinical to clinical studies mid way through year 3. Each survey was sent out twice in the late spring of 2011, with a 2-week delay between each message. No honorarium was offered.

The questionnaire was constructed by the lead researcher and was reviewed by an expert panel for content validity and reliability. Questions were derived from previous literature [19,20] and the researcher’s personal experience and that of other informants. Categories of medical apps enquired upon (Appendix A and Appendix B, question 7) were derived from survey data collated by Garritty et al. [21]. The questionnaire was piloted within one hospital and altered accordingly.

The questionnaire collected data on the following areas: the numbers who owned a smartphone; type of smartphone; the number of medical apps owned and which were most useful; the medical environment in which the smartphone was used; and how often apps were actually referred to during working/educational hours. An open text entry box allowed respondents to discuss any further issues arising from medical related smartphone use.

All numerical data were entered and analysed using the Statistical Package for Social Sciences (SPSS version17), initial descriptive statistics were undertaken and inferential analyses were performed using the non-parametric Chi square test and Fisher Exact tests as appropriate.

Open ended responses from the surveys were initially coded and organised into key themes by the lead author, then verified by the co- authors to support rigour of analysis , trustworthiness and reliability in the interpretation of the data [22]. Quotations illustrating the key themes were later selected and used alongside literature evidence to illustrate the key issues (student (s) or doctor (d) participant number are included to show representativeness and extent of the contributions).

## Results

Findings from each survey are presented separately; where applicable common data is presented together enabling comparisons between surveys groups to be made.

### Junior doctors

Of the junior doctors surveyed 131 answered the questionnaire, out of a possible cohort of 601 employed foundation level trainees; equating to a return rate of 21.8% (131/601). The male to female split was 39.7% (n=52/131) and 60.3% (n=79/131) respectively. Of the 131 participants, 49.6% (n=65/131) were Foundation Year 1 (FY1) trainees, and 50.4% (n=66/131) were Foundation Year 2 (FY2) trainees. Overall, some returns were received from all 8 hospitals within the targeted health region.

Of those junior doctors responding 74.8% (n=98/131) owned a smartphone, with the most popular model being an iPhone, 68.4% (n=67/98), 17.3% (n=17/98) owned a Google android platform smartphone, and a further 14.3% (n=14/98) owned ‘other’ smartphones.

**Figure 1:**
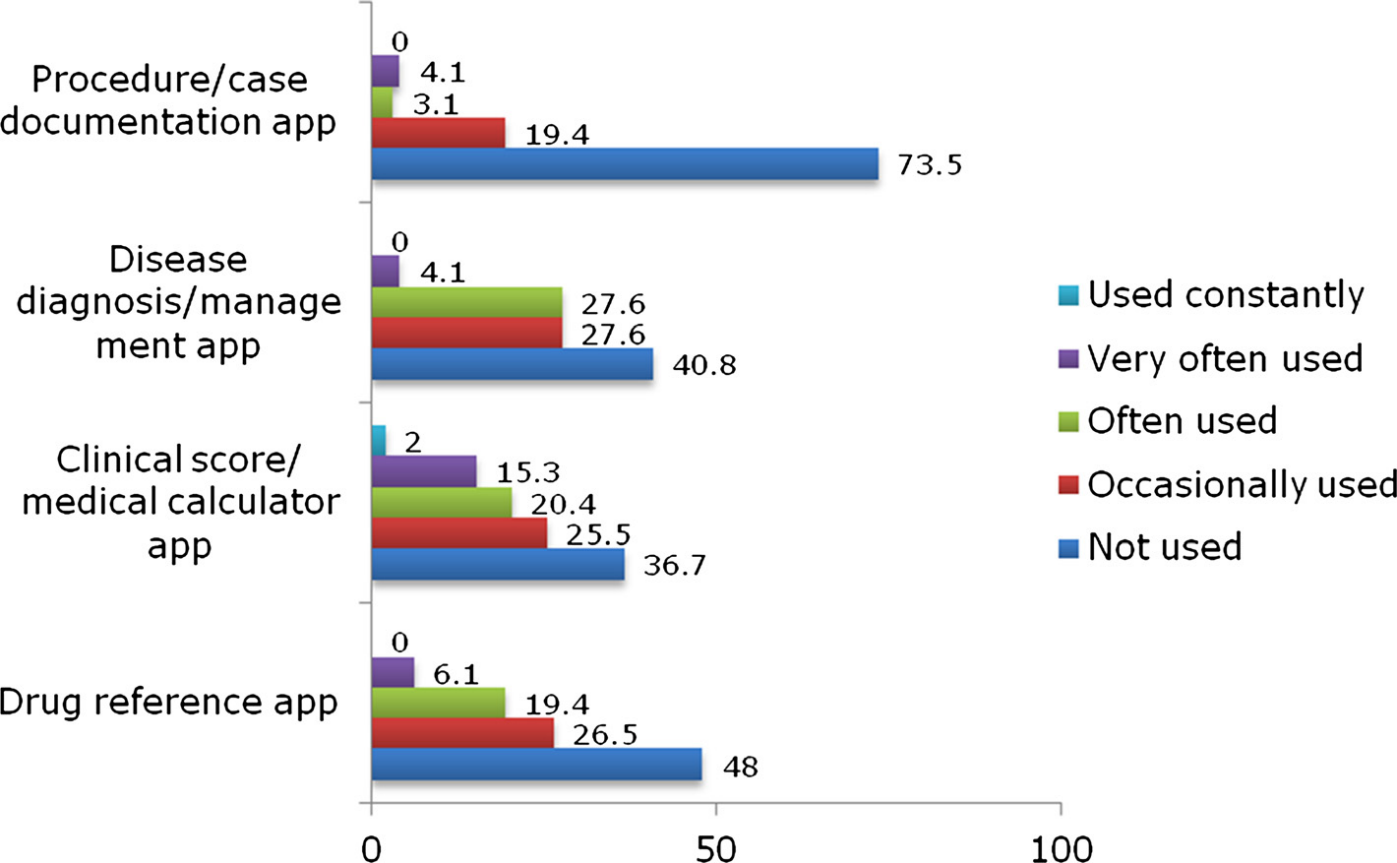
Percentage frequency of use of different categories of medical apps within the junior doctor group (n=98).

The number of medical smartphone apps downloaded by respondents is displayed in Table 1. 74 smartphone owners had apps, 80% of these were iPhone users, 12% had Google android phones and 7% had ‘other’ smartphones. Frequency of app usage is displayed in Table 2, with time spent daily using apps displayed in Table 3. Figure 1 details the type of medical app, and how often these were used by junior doctors.

**Table 1:** Percentage of medical students and junior doctors owning medical related smartphone apps

**Question response**	**Medical student cohort (n=203)**	**Junior doctor cohort (n=98)**
**No**	20.2% (41)	24.5% (24)
**Yes – 1–5 apps**	52.2% (106)	51.0% (50)
**Yes – 6–10 apps**	16.7% (34)	20.4% (20)
**Yes – 11–15 apps**	5.9% (12)	3.1% (3)
**Yes – 16–20 apps**	1.5% (3)	1.0% (1)
**Yes – 21–25 apps**	1.5% (3)	0.0% (0)
**Yes – 26–30 apps**	1.0% (2)	0.0% (0)
**Yes – 30+ apps**	1.0% (2)	0.0% (0)

**Table 2:** Frequency of use of medical related apps within medical student and junior doctor groups

**Question response**	**Medical student cohort**		**Junior doctor cohort (n=98)**
	**Clinical attachment (n=137/203)**	**Medical school education (n=160/203)**	
**Several times a day**	22.6% (31)	14.4% (23)	14.3% (14)
**Once or twice a day**	19.0% (26)	20.0% (32)	15.3% (15)
**2-3 times a week**	19.7% (27)	17.5% (28)	20.4% (20)
**Once a week**	13.1% (18)	14.4% (23)	6.1% (6)
**Rarely used**	8.8% (12)	18.1% (29)	16.3% (16)
**Never used**	16.8% (23)	15.6% (25)	27.6% (27)

**Table 3:** Daily use, in minutes, of medical related apps within medical student and junior doctor groups

**Question response**	**Medical student cohort**		**Junior doctor cohort (n=98)**
	**Clinical attachment (n=137/203)**	**Medical school education (n=148/203)**	
**None**	24.1% (33)	12.2% (18)	27.6% (27)
**1-10 minutes**	27.0% (37)	27.0% (40)	30.6% (30)
**11-20 minutes**	13.9% (19)	21.6% (32)	27.6% (27)
**21-30 minutes**	18.3% (25)	8.9% (13)	11.2% (11)
**31-40 minutes**	9.5% (13)	10.1% (15)	2.0% (2)
**41-50 minutes**	2.9% (4)	6.7% (10)	0.0% (0)
**51-60 minutes**	3.7% (5)	4.1% (6)	1.0% (1)
**60+ minutes**	0.7% (1)	9.5% (14)	0.0% (0)

When questioned about the willingness to access an app specific to their hospital 74.8% (n=98/131) answered yes, they would be willing to use this specific app.

### Medical students

In total 257 medical students answered the questionnaire, out of a possible cohort of 1,706 registered undergraduate students; equating to a return rate of 15.0% (257/1706). The male to female split was 45.1% (n=116/257) and 54.9% (n=141/257) respectively. The distribution of respondents within each year of study were 15.1% (n=39/257) in year 1, 13.2% (n=34/257) in year 2, 6.6% (n=17/257) in year 3 pre-clinical studies, 12% (n=31/257) in year 3 clinical studies, 21.8% (n=56/257) in year 4 clinical studies, and 31.1% (n=80/257) in year 5 clinical studies. When examining pre-clinical and clinical medical students there were approximately equal numbers returned (48.2% and 52.9% respectively).

Of the medical students surveyed 79% (n=203/257) owned a smartphone, with the most popular model being an iPhone, 56.6% (n=115/203) and a further 18.7% (n=38/203) owning a Google android platform smartphone. The number of medical smartphone apps owned by each student is displayed in Table 1. Approximately the same number of males as females owned a smartphone, but males were significantly more likely to own apps (FE=5.43, df=1, p<0.05). iPhone owners were also significantly more likely to own apps (Chi sq=19.68, df=4, p<0.001). 76% of pre-clinical students (n=68/89) and 80% (n=135/168) of students in their clinical years owned a Smartphone.

Students were asked to comment on the purpose of app usage. This was divided into two sections; educational for revision and learning, and clinical for ward and clinic environment. Overall, app usage for educational purposes was most popular (78.3%), with 39.9% using apps for clinical purposes. Within the educational section, revision and learning equated to 73.2% (n=123/168) and 83.3% (n=140/168) respectively. Within the clinical section, ward and clinic environment equated to 42.9% (n=72/168) and 36.9% (n=62/168) respectively. Very few pre-clinical students used apps to support their limited clinical activities in contrast to those in their clinical years, but both groups used apps for education (Chi sq=38.68, df=3 p<0.001).

**Figure 2:**
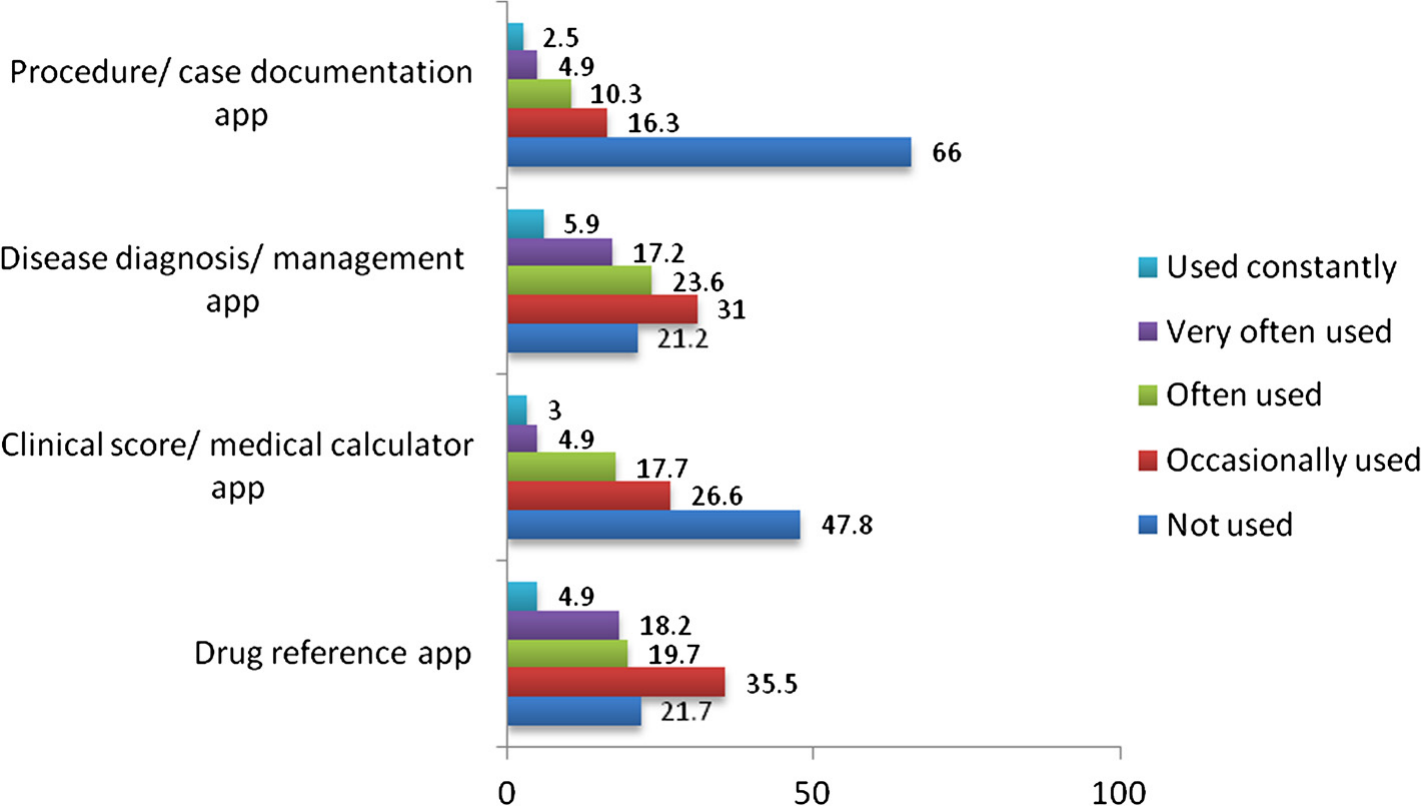
Percentage frequency of use of different categories of medical apps within the medical student group (n=203).

Frequency of app usage is displayed in Table 2, with time spent daily using apps displayed in Table 3. Figure 2 details the type of medical app, and how often these were used by students. When questioned about the willingness to access an app specific to their medical school 96.1% (n=197/203) answered yes, they would be willing to use this specific app.

### Open text data analysis

62.3% (n=160/257) of medical students and 66.4% (n=87/131) of junior doctors chose to answer within the open text entry boxes. Two key themes emerged from this open text data, that of ‘future app development’ and the ‘negative aspects of smartphone use in the clinical environment’; both are illustrated below.

### Future app development

Respondents were keen to state what future apps they would like to see developed, although there was a clear difference between students and doctors. Students desire apps to integrate their timetable, and lecture/module objectives into a handheld source, along with a quiz/test app (Table 4). They want to check ‘*where they should be*’ and ‘*what they should be learning*’, on the go without having to stop to use desktop computers.

**Table 4:** **Type of app listed as desirable by medical student and junior doctor population**, **with number of respondents indicated**

**Respondent group**	**Type of APP**	**Number**
Medical student		
	Timetable linked App	91
	Objectives for lectures/modules	72
	Logbook	52
	Revision note App	38
	Hospital App	18
	University ‘to do list’ App	11
Junior Doctors		
	Antibiotic formulary App	52
	Hospital disease management guideline App	47
	Rota and annual leave App	34
	Acute medical/surgical conditions App	26
	Electronic patient record App	19
	On-call contact details App	8

"“I believe smartphone use is ideal for medical students as it allows easy access to material within seconds, which would take longer searching a textbook. If the university had an app it would assist in accessing lecture notes and enhance student learning” (s13)"

"“Revising using an app is easier, in a car or plane and on holidays” (s22) "

Doctors however, are more concerned about accessing clinical information at point-of-care (Table 4); ‘*what to prescribe*’ and ‘*how to treat an illness*’.

"“I would use an app with clinical guidelines that are hospital specific, including management advice for common conditions. This would be very useful on a busy ward because with only a few computers it is difficult to get a monitor when they are needed for urgent clinical work” (d6)"

"“Please make a hospital app this will save so much time, we can actually treat patients and not spend all day sat at a computer!!” (d19)"

#### Negative aspects of smartphone use in the clinical environment

Several recurring negative themes surrounding smartphone utilisation emerged. Among both students (26 responses) and doctors (15 responses) the issue of cost, of both smartphones and apps, was a common theme.

"“I find it very annoying that so much emphasis is placed on smartphones, as not everyone can afford one” (s45)"

"“I would love to use the BNF* on the iPhone but it is very expensive!” (d29)"

"“It is difficult to find free or cheap apps that contain enough material to last longer than a few uses, before all the material has been seen” (s26)"

*(BNF - British National Formulary: a nationally approved drug dosing guide)

Specific to doctors’ open text data (17 responses) was the theme of the appearance to others of using their smartphone in the workplace.

"“I think it appears extremely rude both to patient and colleagues to appear to be looking at your phone whilst on the ward, as it is usually misinterpreted as checking texts or emails” (d8)"

"“The culture of looking lazy or uninterested by playing on your phone needs to be overcome. Once patients and consultants realise the phone use is work related I will feel more comfortable” (d31)"

## Discussion

This study represents primary research for the UK, the first formal survey to investigate smartphone ownership and usage in a junior doctor and medical student population; this data adds valuable insight to an evolving UK healthcare system. This research is timely since the UK Department of Health have recently published a Framework for Technology Enhanced Learning [23] in which the potential for the use of innovative technologies including smartphones is recognised:

"‘Innovative educational technologies, such as e-learning, simulation and smartphones, provide unprecedented opportunities for health and social care students, trainees and staff to acquire, develop and maintain the essential knowledge, skills, values and behaviours needed for safe and effective patient care.’ (pg 6) "

It is therefore important to capture patterns of smartphone use in healthcare education and clinical practice in order to develop and recommend appropriate learning materials and activities for delivery on smartphone platforms.

### Numbers owning a smartphone and frequency of medical app use

A systematic review by Garritty et al. concluded that PDA usage among healthcare providers varied from 45% to 85%, noting younger physicians or residents and those in large hospitals were more likely to use a PDA [21]. Although this data was derived largely from the United States, with no data from the UK, our results do corroborate their findings and demonstrate a large number of respondents owning and using medical apps on their smartphone. A hospital based survey undertaken by Dasari et al. who questioned British anaesthetists across a breadth of training grades, reported that 59% owned an iPhone. 80% of those owning an iPhone actively used medical apps, with 60% using them for clinical activities and 47% for educational activities [19]. In comparison, our data points towards higher medical app usage rates within a comparable doctor group, with 72.4% of doctors using medical apps, to varying degrees, during clinical activities (Table 2).

Within a US medical student population, Grasso et al. reported 52% of medical students using handheld computers, however displaying a clear divide between pre-clinical and clinical years (28% and 76% respectively) [20]. In contrast our data displayed equal smartphone ownership within pre-clinical and clinical years (76% and 80% respectively), with overall app usage as high as 83.3% (for educational purposes). This change should be interpreted cautiously, taking into account the inherent difference between a handheld computer and a smartphone, and the varying postgraduate structure of US and UK medical degrees.

Within the student group, educational use of apps starts in pre-clinical years and appears to follow into clinical years, with both year groups using apps more for educational than clinical purposes. However there appears to be no pattern relating to frequency and time spent using apps specific to clinical and educational environments (Tables 2 and 3). If we acknowledge the touted benefits of handheld technology in the clinical environment for clinicians, we would expect a higher frequency of app use in a clinical environment also within a student population. Students are using apps to learn at all stages of their studies, and this factor should be taken into consideration when designing or developing further apps in these settings.

Comparing student and doctor groups: smartphone ownership and frequency of app use is similar, however time spent daily using apps is reduced within the doctor group. The majority of doctors reported using apps daily for between 1 and 20 minutes, with very few using apps for longer than 30 minutes (Table 3). In contrast to students, who display a more even spread, especially for medical school educational activities. It is unclear if this pattern is due to external time pressures of the environment within which the app is used (i.e. a busy hospital setting for doctors), or the true intention or need of students to use apps for longer. It suggests doctors are using apps for quick reference as opposed to prolonged educational reading.

### Number and type of medical apps used or owned

The findings suggest that students and doctors are selective about the medical apps they download and/or purchase, the majority owned 1 to 5 apps which they used on a regular basis. Similar to previous studies, the most frequently used apps in the student cohort were those detailing medication/drug reference [20] and those involved with disease diagnosis/management (Figure 2). In slight contrast, the doctor cohort used clinical scoring apps more often (Figure 1). The nature of apps used reflects the manner in which smartphones appear to be utilised. In the clinical environment smartphones are often used at point of care [2]; thus for a doctor, apps that increase efficiency by saving time and allow ‘mobile’ rapid decision making are going to be popular [1]. For a student, disease diagnosis/management related apps are more likely to fullfill their educational needs when compared to more simple clinical scoring/calculator type apps.

In both groups apps used for procedure/case documentation scored very low, with over half of participants not using these apps (Figures 1 and 2). This is perhaps attributable to the lack of apps currently available in this area, and that for an app of this nature to be regularly used it would likely need to be linked to the relevant medical school or regional postgraduate healthcare organisation. The finding that both medical students and junior doctors own relatively few medical apps, but use them often, has key ramifications for the development of future organisational Medical School or Hospital linked apps. For an app to be used by a medical student or junior doctor it clearly needs to be of high quality and be fit for purpose to avoid user neglect. On the other hand, as medical students and junior doctors appear to own a relatively small number of apps, the chance of integrating such an app into regular use is increased.

### Future app development

From the analysis of open text entry data, there is a clear trend that both students and doctors want apps linked to their respective organisations. For students the desired purpose of these appears to be to receive administrative information relating to timetabling and lecture content, and secondly the need for revision and quiz type apps. This does align with previous studies describing using mobile devices to enhance competency sign offs [17], and student case logbooks [18]. No studies have explored the benefit of incorporating lecture timetabling and lecture objectives into a smartphone format.

Doctors are concerned about clinical information relating to patient management and care, with administrative apps relating to rotas and annual leave receiving less attention. Using a search on the Apple appstore, we were able to find relatively few doctor orientated UK hospital associated apps (this method does not take into account apps distributed directly to workers through Apple’s commercial app license). Among these were a Microbiology app [24], a Paediatric Drug dosing app [25] and a Thromboprophylaxis app [26]. This suggests that the needs of British junior doctors, relating to smartphone apps, are currently not being fully addressed. As an example of full smartphone hospital integration; the Samsung Medical Center in Seoul, Korea has launched a smartphone app that allows doctors access to both inpatient records and lab results [27]. They provide evidence of a positive response from doctors, with regular use of this app during working hours. With the evidence that our doctor group reported regular use of disease diagnosis/management apps and drug reference apps (both areas for which hospitals develop individual clinical guidelines), the possibility of these guidelines being linked to smartphone apps specific to UK hospital sites should be explored in future research. This would remove the medico-legal risk of hospital staff using smartphone based clinical resources not referenced or reviewed by accepted authorities.

### Negative aspects of using smartphones

Concern about the cost of a smartphone and medical apps is an important finding among both student and doctor groups. There are policy implications around the expectation that all students and doctors should be expected to have a smartphone to support their education or professional practice. Furthermore what policies should be in place to prevent discriminating against those, who for whatever reason, choose not to endorse this technology?

One obvious solution to this problem of incurred costs for the individual is for the organisation to provide smartphones and free access to apps. This solution is currently being evaluated within the Wales Deanery (UK regional healthcare organisation) among all Foundation Doctors (American ‘Intern’ equivalent) [28], with the results not yet published in full. As our data points to the majority of students and doctors owning smartphones, it could be argued that the cost of scoping, designing and developing suitable high quality apps and advertising the utility of free apps that are available, is the more pertinent issue to be immediately addressed.

Not to be overlooked are the personal concerns of doctors relating to the experience of using smartphones in a clinical environment and the effect on the doctor-patient relationship/communication, and the negative connotations associated with doing so. This re-iterates physician concerns regarding patients’ perceptions of mobile phone use in the clinical environment as previously identified [29] and clearly requires further in-depth qualitative investigation. Use of smartphones in clinical areas may well require a shift in service user attitudes towards this mobile technology. Overcoming this barrier will require an investment in doctor education at all grades, and a definite policy from organisations to endorse smartphone technology.

### Limitations

This study focused on students and young doctors, both are groups which may be more ‘IT savvy’ and likely to use smartphone technology. Higher trainees and consultants were not questioned within this study. The chosen site for conducting this study comprised a large population fitting the inclusion criteria however the response rate was relatively low (15% and 21.8%, in student and doctor groups respectively), and limits the level to which these results can be generalised to other similar groups. The number of responses received is comparable to similar regional surveys of this kind [20]; in a published review of hand held device use by healthcare providers, the average response rate in 4 of the most up-to-date published studies was 27.6% [21], with one study using an online questionnaire (as in our study) obtaining an 11% response rate. Many study articles in this review did not report a response rate.

The increased likeliness of smartphone users to answer a survey related to smartphone use is noted as a definite source of non-response bias and voluntary response bias and using an online questionnaire may have contributed to this study design limitation. However the prevalence of smartphone ownership was only one objective of the survey and it does provide a snapshot at just one point in time providing a useful benchmark for future studies.

This survey was intentionally conducted during university term time to ensure a representative student response

## Conclusion

Smartphones will soon be universally owned among the medical profession and offer a real opportunity to impact on the efficiency of working practices and patient care with minimal capital outlay for healthcare organisations. High levels of smartphone ownership and the intuitive and user-friendly interfaces associated with many apps suggest that the traditional ‘barriers’ to implementing new technologies are no longer applicable [30]. Ultimately large organisations may remain sceptical to invest time and money into smartphone implementations if they cannot see a clear benefit to patient care and safety, and evidence to support this.

We report an apparent rise in smartphone ownership and medical app usage among medical student and doctor groups, with similar levels of smartphone ownership and patterns of medical app use when comparing these two groups.

However, a differing opinion between these groups exists, regarding the type of apps desired to facilitate future smartphone usage. The “take home message” is that junior doctors and medical students are overwhelmingly enthusiastic to endorse organisational associated apps that help their learning and work activities. However, organisations and developers should be mindful of the negative issues surrounding medical app use in the clinical environment from both clinicians and service users. Future work should focus not only on appropriate app development but also on the perceptions of health care professionals and users on the use of mobile technologies in clinical areas.

## Appendix A

Questionnaire 1: Survey distributed to Foundation Doctors.

1. Please state your gender:

a) Male

b) Female

2. Please state your current position and job rotation: (choice of either ‘FY1’ or FY2’ chosen for one of the below options)

a) Medicine

b) Surgery

c) GP

d) Anaesthetics

e) Psychiatry

f) Other

3. Please state your current hospital of employment:

a) Queens Medical Centre Nottingham

b) Nottingham City Hospital

c) Royal Derby Hospital

d) King’s Mill Hospital Mansfield

e) Lincoln County Hospital

f) Grantham Hospital

g) Pilgrim Hospital Boson

h) Chesterfield Royal Hospital

4. Do you own an application smartphone?

a) No

b) Yes – iPhone

c) Yes – Google Android

d) Yes – other smartphone

5. Concerning your smartphone, do you own medical related applications?

a) No

b) Yes – 1–5

c) Yes – 6–10

d) Yes – 11–15

e) Yes – 16–20

f) Yes – 21–25

g) Yes – 26–30

h) Yes – 31+

6. Please estimate the frequency, during working hours, you utilise medical applications on your smartphone:

a) Several times a day

b) Once or twice a day

c) 2–3 times a week

d) once a week

e) rarely used

f) never used

7. In relation to the following types of applications, please indicate how often you use them to help you with your clinical activities: (choice of ‘not used’, ‘occasionally used’, ‘often used’, ‘very often used’ and ‘used constantly’ for each of below)

a) medication formulary/drug reference

b) clinical score systems/medical calculator

c) disease diagnosis/management

d) procedure documentation

e) web access

f) email access

g) calendar

h) password storage

i) other (please detail in comment box)

8. Please estimate the time you spend per day (in minutes) using smartphone applications related to clinical activities:

a) none

b) 1–10

c) 11–20

d) 21–30

e) 31–40

f) 41–50

g) 51–60

h) 61+

9. Would you utilise a smartphone app specific to your Medical School?

a) Yes

b) No

10. Please detail any further comments you have regarding your use of medical related smartphone applications in the clinical environment:

Which specific apps would you recommend? What features do you find most useful in a medical related app?

If so, why do you find your smartphone useful at work?

Do you use any other portable digital assistant/handheld computer at work?

(free text entry box provided)

## Appendix B

Questionnaire 2: Survey distributed to Medical Students

1. Please state your gender:

a) Male

b) Female

2. Please state your current year of study:

a) 1^st^ year

b) 2^nd^ year

c) 3^rd^ year pre-clinical

d) 3^rd^ year clinical

e) 4^th^ year

f) 5^th^ year

3. Do you own an application smartphone?

a) e)No

b) f)Yes – iPhone

c) g)Yes – Google Android

d) h)Yes – other smartphone

4. Concerning your smartphone, do you own medical related applications?

a) No

b) Yes – 1–5

c) Yes – 6–10

d) Yes – 11–15

e) Yes – 16–20

f) Yes – 21–25

g) Yes – 26–30

h) Yes – 31+

5. Please indicate how you use medical related app: (you may choose more than one answer)

a) Education – revising

b) Education – learning

c) Clinical – ward environment

d) Clinical – clinic environment

6. Please estimate the frequency you utilise medical applications on your smartphone during clinical attachment compared to medical school educational time. (one of the below options chosen for the categories: ‘clinical attachment’ and ‘medical school education’)

a) Several times a day

b) Once or twice a day

c) 2–3 times a week

d) once a week

e) rarely used

f) never used

7. In relation to the following types of applications, please indicate how often you use them during educational and/or clinical hours: (choice of ‘not used’, ‘occasionally used’, ‘often used’, ‘very often used’ and ‘used constantly’ for each of below)

a) medication formulary/drug reference

b) clinical score systems/medical calculator

c) disease diagnosis/management

d) procedure documentation

e) web access

f) email access

g) calendar

h) password storage

i) other (please detail in comment box)

8. Please estimate the time you spend per day (in minutes) using smartphone applications related to clinical and educational activities: (one of the below options chosen for the categories ‘clinical’ and ‘education’)

a) none

b) 1–10

c) 11–20

d) 21–30

e) 31–40

f) 41–50

g) 51–60

h) 61+

9. Would you utilise a smartphone app specific to your Medical School?

a) Yes

b) No

10. Please detail any further comments you have regarding your use of medical related smartphone applications in the clinical environment:

What characteristics would you find useful in a Medical School linked app?

Which specific apps would you recommend?

What features do you find most useful in a medical related app?

(free text entry box provided)

## Competing interests

The authors declared that they have no competing interest.

## Authors’ contributions

KP conceived the idea and managed the data collection process. HW and KW were involved in the development and refinement of the data collection tools, interpretation and presentation of the data and carried out the data analysis. All the authors were involved in drafting and redrafting the manuscript and revising it critically for intellectual content. All authors read and approved the final manuscript.

## Pre-publication history

The pre-publication history for this paper can be accessed here:

http://www.biomedcentral.com/1472-6947/12/121/prepub
